# Dual-targeted lung cancer therapy *via* inhalation delivery of UCNP-siRNA-AS1411 nanocages

**DOI:** 10.20892/j.issn.2095-3941.2020.0416

**Published:** 2021-08-24

**Authors:** Yu Han, Yuming Yang, Qiuyang Sun, Bin Li, Caixia Yue, Yanlei Liu, Jesús M. de la Fuente, Daxiang Cui

**Affiliations:** 1 Institute of Nano Biomedicine and Engineering, Key Laboratory for Thin Film and Microfabrication Technology of the Ministry of Education, Shanghai Engineering Research Center for Intelligent Diagnosis and Treatment Instrument, Department of Instrument Science & Engineering, School of Electronic Information and Electrical Engineering, Shanghai Jiao Tong University, Shanghai 200240, China; 2 National Engineering Center for Nanotechnology, Collaborative Innovational Center for System Biology, Shanghai 200241, China; 3 Department of Neurosurgery, Xinhua Hospital, Shanghai Jiaotong University School of Medicine, Shanghai 200092, China; 4 School of Biomedical Engineering, Shanghai Jiao Tong University, Shanghai 200240, China; 5 Instituto de Nanociencia de Aragon (INA), Universidad de Zaragoza, Zaragoza 50018, Spain

**Keywords:** Nanomaterials, VEGF siRNA, lung cancer, gene therapy, siRNA delivery

## Abstract

**Objective:**

Although great progress has been made in the field of siRNA gene therapy, safe, efficient, and targeted delivery of siRNA are still major challenges in siRNA therapeutics.

**Methods:**

We developed an up-conversion nanoparticle-based nanocage system. This system protected the siRNA from being degraded by nucleases in organisms and selectively delivered the siRNAs to the tumor sites, due to modifications of targeted molecules on the surfaces of nanocages and local inhalation.

**Results:**

The siRNAs delivered by the up-conversion nanoparticle nanocages were protected from degradation in transit to the tumor sites, where they accumulated. Compared with the passive target and control groups, the up-conversion nanoparticles based on the nanocage system showed a tumor suppressive effect after approximately 3 weeks of treatment.

**Conclusions:**

The up-conversion nanoparticle nanocages efficiently delivered vascular endothelial growth factor siRNAs to tumor sites. Mice with lung tumors treated with tumors targeting up-conversion nanoparticle nanocages showed steady body weight changes, high tumor inhibition ratios, and longer survival times.

## Introduction

Gene therapy is the use of genetic engineering technology to introduce normal genes into the cells of patients to correct defective genes and eradicate diseases. Vascular endothelial growth factor (VEGF), which was identified in the 1980s, is recognized as an essential regulator of normal and abnormal blood vessel growth. The recent approval of bevacizumab by the USFDA as a first-line therapy for metastatic colorectal cancer confirmed that VEGF was a key mediator of tumor angiogenesis, and that blocking angiogenesis was an effective strategy to treat human cancers^1^. The ability of transfer genes to target tissues and organs has been proven to be one of the best methods for precise drug therapy^2,3^. Vectors currently used in gene therapy involve viral vectors and non-viral vectors. The most commonly used viral vectors contain retrovirus, adenovirus, adeno-associated virus, herpes simplex virus, or lentivirus^4^. Due to cytotoxicity, immunogenicity, limited vector capacity, and difficulty in increasing the viral titer of viral vectors, non-viral gene therapy vector systems have attracted increasing attention^5^. Non-viral vectors are tools used for gene transfer mediated by physical and chemical methods. Compared with viral vectors, their outstanding advantages involve low toxicity, high safety, simple preparation, and a relatively larger capacity for carrying foreign genes. Nude DNA, liposomes, polymers^6^, and molecular conjugates are commonly used as non-viral vectors^6–9^. However, the low transfection efficiency, short gene expression time, ease of scavenging by tissues, and poor targeting ability limit their applications. A suitable gene delivery system that could protect nucleic acids from degradation and selectively deliver sufficient amounts of siRNAs to target sites is therefore needed.

In recent years, nanoparticles have been used for drug delivery, and now are widely used for tumor imaging and therapy^10–12^. There are also many nanoparticle-based vesicle systems for miRNA delivery^9^, such as lipids^13^ or polymeric-based nanoparticles^14–18^, bio-inspired nanovehicles^19^, and inorganic nanomaterials^20–22^. These studies showed the possibility of using nanoparticles as siRNA carriers, but siRNA is degraded by various enzymes in serum during the delivery process. These vesicles cannot deliver sufficient amounts of siRNAs, because most of the siRNAs have been degraded during transit to the target sites.

**Figure 1:**
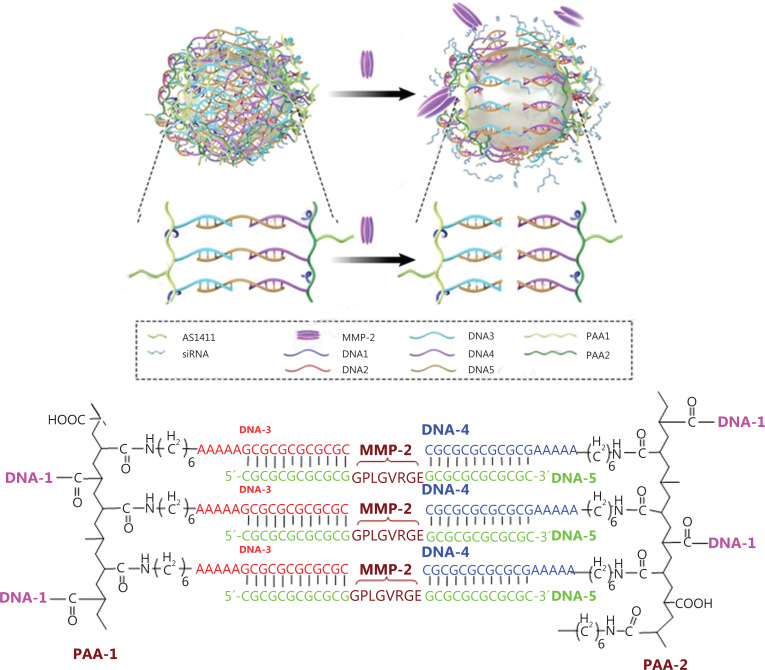
Schematic illustration of the UCNP nanocage structure and tumor responsive gene release mechanism.

In this study, we designed an up-conversion nanoparticle (UCNP)-based nanocage system. The UCNPs were selected as the gene delivery vehicle because of their narrow emission bandwidth, long luminescence lifetime, adjustable emission spectrum, high light stability, relatively low cytotoxicity, and more importantly, zero background in the imaging process, resulting in very high imaging sensitivity. Polyacrylic acids (PAAs) were selected to construct the surface of the nanocage due to their large amounts of carboxylic acid groups, which facilitated conjugation of single strand DNAs using amidation. In addition, the high water solubility and negative surface charge of PAAs greatly improved the biocompatibility of the UCNPs. A thermal decomposition method was used to synthesize UCNPs. Carboxyl groups modified double strand DNAs, which acted as connectors of the core (UCNPs) and the surface of the nanocages. Detailed construction of the UCNP nanocage is shown in **Figure 1**. PAAs were divided into 2 groups. One group (PAA-1) was conjugated with NH_2_-modified DNA-1 (AS1411) and DNA-3 using an amidation reaction. Similarly, the other PAA group (PAA-2) was conjugated with NH_2_-modified DNA-1 and DNA-4. Carboxyl groups modified DNA-2, which were first modified on the surface of the UCNPs. The PAA-1 and PAA-2 were conjugated on the surface of UCNPs using the hybridization of DNA-1 and DNA-2, because DNA-2 was the complementary antisense strand of DNA-1. The UCNP nanocages were finally closed using complementary base pairing of DNA-3 and DNA-4 with DNA-5. To achieve a tumor responsive drug release, DNA-5 was modified with a linker using a MMP-2 cleavable peptide. The siRNAs were loaded inside the cage, which was protected from being degraded by enzymes during transit to the target site. Once the UCNP nanocages arrived at the tumor tissue, the overexpression of the MMP-2 enzyme triggered the release of siRNAs.

## Materials and methods

### Synthesis and characterization of UCNP nanoparticles

At room temperature, 4 mmol of CF_3_COONa and 2 mmol (total amounts) of Ln (CF_3_COO)_3_ (Ln: 78 mol% Lu + 20 mol% Yb + 1.6 mol% Er + 0.4 mol% Tm) were added to 20 mL oleyl amine (OM) in a 100 mL 3-neck round bottom flask. Next, the reaction solution was directly heated to 120 °C to remove water and oxygen, with vigorous magnetic stirring in Argon for 1 h. At this point, the reaction mixture was a transparent solution. The solution was then heated to 330 °C under Argon at a rate of 10 °C per min and maintained at this temperature for 2.5 h. After the reaction was completed, 10 mL of cyclohexane was poured into the solution at room temperature. The resultant mixture was centrifugally separated (10 min every time at 20 °C), washed with cyclohexane and chloroform 3 times, and dried under vacuum at room temperature overnight.

### The surface treatment of UCNPs

A total of 300 nM UCNP nanoparticles was incubated with a 800-fold excess of carbonyl modified DNA-2 and siRNA (DNA-2: siRNA = 1:10 molar ratio) in 0.5× TBE ultrasound for 30 min. Centrifugation (8,000 rpm for 40 min) was used to remove salt and free DNA. The most efficient siRNA duplex for mouse VEGF with the sequence 5′-CCCACAUACACACAUAUAUUU (sense) and 3′-UUGGGUGUAUGUGUGUAU-AUA (antisense) was selected from *in vitro* screening and was used for subsequent *in vitro* and *in vivo* tests.

### UCNP nanocage construction

PAA functionalization with oligonucleotides was performed by carbodiimide chemistry assisted by the N-hydroxysuccinimide coupling reaction between the carboxylated PAA and the primary amine groups of the oligonucleotides. PAA-Group1 was conjugated using 5-fold molar ratios of DNA-1(AS1411) and DNA-3. The resultant products were purified by dialysis for 72 h. PAA-2 was conjugated with 5-fold molar ratios of DNA-1(AS1411) and DNA-4. DNA-2 oligonucleotide derivatized UCNP nanoparticles were incubated with PAA-1, PAA-2, and ssDNA-5 in 0.5× TBE overnight. The final UCNP nanocages were purified by centrifugation at 12,000 × *g* 2–3 times for 30 min in 0.5× TBE. To determine the average size, particle distribution, and morphology of the UCNP nanocages, the samples were analyzed using dynamic light scattering and field emission transmission electron microscopy (TEM; JEM-2100F; JEOL, Tokyo, Japan) with 1 mg/mL uranyl acetate used for negative staining. The UCNP nanocage solution was dripped onto a carbon film-supported copper mesh grid and allowed to air dry before being subjected to TEM analysis.

### *In vitro* siRNA release from UCNP nanocages

A dialysis method was used to characterize the release behavior of siRNA from the UCNP nanocages. First, 3 mL of UCNP nanocages were transferred into a dialysis bag with 18,000 molecular weight cut-off. The dialysis bag was then dipped into 50 mL cell culture medium containing 10% serum and incubated at 37 °C with continuous stirring at 200 rpm for as long as 72 h. Then, 0.3 mL of the external cell culture medium was removed for analysis at predetermined time intervals, and an equal volume of dialysis solution was replenished. The amount of Cy5-modified siRNA released was quantified by measuring the fluorescence signal of Cy5. The DOX concentration was measured by high-pressure liquid chromatography.

### Cell culture

The NCI-H889 lung-adenocarcinoma cell line was selected for *in vitro* targeting and cell apoptosis experiments. They were cultured in DMEM with 10% heat inactivated fetal bovine serum (Gibco, Gaithersburg, MD, USA), 100 units of potassium penicillin, and 100 µg of streptomycin sulfate per 1 mL of culture media at 37 °C in 5% CO_2_.

### Cellular uptake and distribution of self-assembled nanodrugs

The NCI-H889 lung adenocarcinoma cells were seeded onto 28 mm glass cover slips for 24 h before being incubated with UCNP nanocages. The cells were then incubated with 100 µg/mL UCNP-siRNA-PAA-AS1411/UCNP-siRNA nanocages at 37 °C for 1 h, then washed twice with phosphate-buffered saline (PBS) and fixed with a 4% paraformaldehyde solution for 30 min at room temperature. The nuclei were then stained with Hoechst 33342 (10 µg/mL) at 37 °C for 15 min, and the slides were rinsed 3 times with PBS. A confocal laser scanning microscope (CLSM; TCS SP8; Leica, Wetzlar, Germany) was used to detect the cellular uptake and distribution of UCNP nanocages in NCI-H889 cells. To visualize the cellular distribution of UCNP nanocages using TEM, NCI-H889 lung adenocarcinoma cells were incubated with 500 µg/mL UCNP-siRNA-PAA-AS1411 nanocages for up to 48 h and then fixed with 2.5% glutaraldehyde for more than 1 h. The samples were then sent to Shanghai Normal University (Shanghai, China) for TEM analysis.

### *In vivo* targeted delivery of UCNP nanocages

The NCI-H889 lung adenocarcinoma cells were selected to construct the lung cancer orthotopic murine model. Five-week-old female BALB/c nude mice were purchased from the Experimental Animal Center of Shanghai Jiao Tong University (Shanghai, China) and maintained in individually ventilated cages on a 12 h light/12 h dark cycle, at 21–23 °C and 40%–60% humidity with free access to food and water. All procedures were performed in accordance with protocols approved by the Institutional Animal Care and Use Committee of Shanghai Jiao Tong University (Approval No. 201902043). Animals were allowed to acclimate for approximately 5 days before any procedures. The lung cancer orthotopic murine model was established by subcutaneously injecting NCI-H889 cells into the lungs of 6-week-old BALB/c nude mice after making a 0.5 cm cut under the front leg. Six BALB/c nude mice bearing NCI-H889 lung tumors in each group were treated with 1 mg/mL UCNP-siRNA-PAA-AS1411 and UCNP-siRNA nanoparticles using the inhalation-mediated local delivery method. The fluorescence distribution was monitored at 1 and 4 h using a small animal *in vivo* imaging system with a 980 nm excitation light source. UCNP nanocage fluorescence was measured at λexcitation = 980 nm, λemission = 801 ± 20 nm; tdTomato-labeled tumor fluorescence, λexcitation = 554 nm, λemission = 581 ± 20 nm. The mice used for the *in vitro* organ imaging were euthanized 4 h after the UCNP nanocages were administered. All animal experiments complied with the ARRIVE guidelines and were conducted in accordance with the National Institutes of Health Guide for the Care and Use of Laboratory Animals (NIH Publication No. 8023, revised 1978).

### Tumor inhibition activity of UCNP nanocages

BALB/c nude mice bearing the NCI-H889 orthotopic murine model of lung cancer were treated with UCNP-siRNA-PAA-AS1411 UCNP nanocages on days 12, 15, 18, 21, 24, 27, 30, 33, and 36. Considering waste during inhalation administration, 1 mL of 1 mg/mL UCNP-siRNA-PAA-AS1411 UCNP nanocages were used. Two groups of orthotopic lung tumor-bearing mice treated with UCNP-siRNA only were used as the control. All experiments included 6 mice per treatment group unless otherwise noted.

### Quantitative PCR

Total RNA from NCI-H889 lung adenocarcinoma cells was extracted using the RNeasy Plus Mini Kit (Qiagen, Hilden, Germany) according to the manufacture’s protocol. The qRT-PCR was performed using the SuperScript™ III One-Step RT-PCR System with the Platinum™ Taq DNA Polymerase Kit (Thermo Fisher Scientific, Waltham, MA, USA). The samples were placed in the preheated thermal cycler, which was programmed with the following thermal cycling procedure: 45–60 °C for 30 min; 2 min at 94 °C; 40 cycles at 15 s at 94 °C, 30 s at 55–66 °C, 1 min at 68 °C; and finally, 1 cycle for 5 min at 68 °C. Glyceraldehyde 3-phosphate dehydrogenase was used as the reference gene.

### Histology and immunohistochemistry

Freshly removed lung tumor tissues were rinsed with sterile PBS, frozen with liquid nitrogen, and sectioned using a cryostat microtome. For the immunohistochemical analyses, lung (tumor) sections were first fixed with 4% paraformaldehyde for 15 min, then treated with 0.25% Triton X-100 for 10 min, and washed with PBS, 3 times. Next, the tissues were blocked in PBST containing 1% bovine serum albumin for 30 min and then washed with PBS, 3 times.

The tissues were then incubated with an anti-vascular endothelial growth factor (VEGF) antibody for 1 h at room temperature, then washed 3 times with PBS. The tissues were then incubated with the secondary antibody (Alexa Fluor 532 goat anti-mouse IgG, 1:250; Invitrogen, Carlsbad, CA, USA) for 1 h at room temperature and subsequently washed 3 times with PBS. The nuclei were then stained with Hoechst 33342 (10 µg/mL) at 37 °C for 15 min and then rinsed 3 times with PBS. All slices were examined by confocal laser scanning microscopy (TCS SP8, Leica).

### Statistical analysis

Differences between groups were examined using Student’s paired *t*-test using SPSS statistical software for Windows, version 17 (SPSS, Chicago, IL, USA). All error bars used in this report represent the mean ± SD of 3–6 independent experiments. Statistically significant *P*-values were indicated in figures and/or captions as ***P < 0.005; **P < 0.01; *P < 0.05. All *in vivo* experiments used 6 mice per treatment group unless otherwise noted.

## Results

### Synthesis and characterization of UCNP nanocages

NaLuF_4_: 20% Yb, 0.4 mol% Tm, 1.6 mol% Er up-conversion nanoparticles were synthesized according to the thermal decomposition method using oleylamine as the surface ligand. As indicated in the scheme, we modified ssDNA-2 with carboxyl groups at the 5′ end, then reacted them with UCNPs using carboxyl group conjugation. The antisense of ssDNA-2 (ssDNA-1) was first reacted with PAA, then double-stranded DNA was formed using the base pair recognition mechanism to construct the supporting structure of the UCNP nanocage. To build a closed cage structure, the PAAs were divided into 2 groups. The first group of PAAs (PAA-1) was modified with ssDNA-1 and ssDNA-3. The second group of PAAs (PAA-2) was modified with ssDNA-1 and ssDNA-4. After equal molar ratios of PAA-1, PAA-2, and ssDNA-5 were added to the ssDNA-2 modified UCNP nanoparticles solution, UCNP nanocages were finally closed through the complementary base pairing of DNA-3 and DNA-4 with DNA-5. To increase the targeting performance, we selected AS1411 as the ssDNA-1 to construct the nanocages. Parts of the ssDNA-1 bound with ssDNA-2 and the others were used to increase the UCNP nanocage targeting ability. In addition, a MMP-2 cleavable peptide was introduced into the DNA-5, which endowed the UCNP nanocages with tumor-responsive gene release properties.

**Figure 2:**
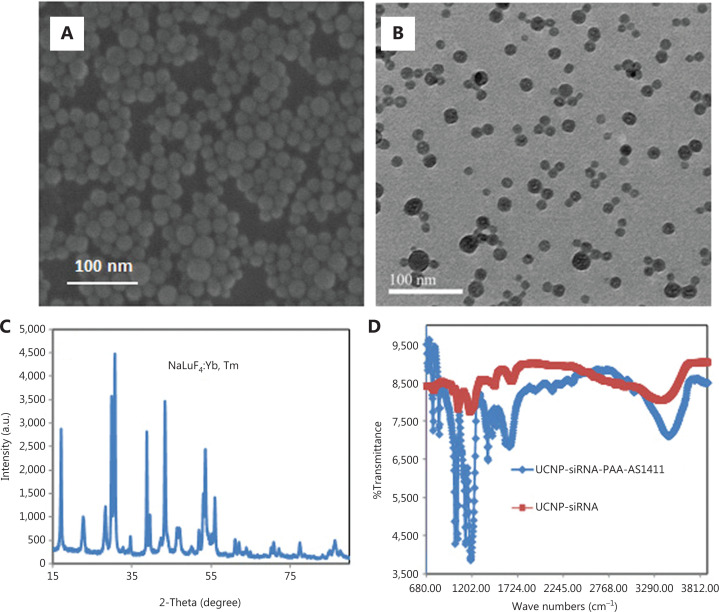
Characterization of UCNP nanocages. (A) Scanning electron microscopy image of UCNP (NaluF4:Yb, Tm) nanoparticles. (B) Transmission electron microcopy of UCNP-siRNA-PAA-AS1411 nanoparticles. (C) X-ray diffraction of NaluF_4_:Yb, Tm nanoparticles. (D) Fourier transform infrared spectrophotometry of UCNP, UCNP-siRNA, and UCNP-siRNA-PAA-AS1411 using ethanol as a solvent.

The oleylamine-modified UCNPs and UCNP nanocages were characterized by X-ray diffraction (XRD), TEM, and Fourier transform infrared spectroscopy (FTIR) (**Figure 2**). TEM images showed that the oleylamine-modified NaLuF_4_:Yb, Tm nanoparticles exhibited a uniform morphology with a diameter of approximately 20 nm, and were easily dispersed in chloroform. The UCNP nanocages also exhibited good dispersity, which showed that controlling the concentration of UCNP nanoparticles in the synthesis process effectively reduced the cross-linking. The XRD pattern of UCNP-PAA nanocages was indexed to the α and β phases of NaLuF_4_. The successful synthesis of UCNP-PAA nanocages was investigated by FTIR spectrophotometry. FTIR spectrophotometry of UCNP-ssDNA showed 2 absorption peaks at 3,380 cm^−1^ and 3,100 cm^−1^, which were the nitrogen hydrogen bonds of primary amines; the peak at 1,710 cm^−1^ was the absorption peak of the keto carbonyl, and 1,400 cm^−1^ and 1,200 cm^−1^ were the absorption peaks of conjugated structures of pyrimidines and carbon oxygen bonds, respectively. From the above analyses, the chemical functional groups present in the UCNP-siRNA-PAA-AS1411 were absent in pure UCNPs, so it was concluded that DNA was successfully linked to the UCNPs.

The average diameter of the UCNP-siRNA-PAA-AS1411 was approximately 30 nm, because PAAs and DNAs were connected to the surface of UCNPs, resulting in an increase of hydrated particle size. In addition, the zeta potentials of UCNPs and UCNP-siRNA-PAA-AS1411 were approximately +12 mV and −12 mV, respectively. The 48 h dynamic light scattering data showed that the particle size of the material in the serum environment showed no obvious change, indicating good stability in the serum environment.

### *In vitro* tumor targeting and siRNA release characterization

**Figure 3:**
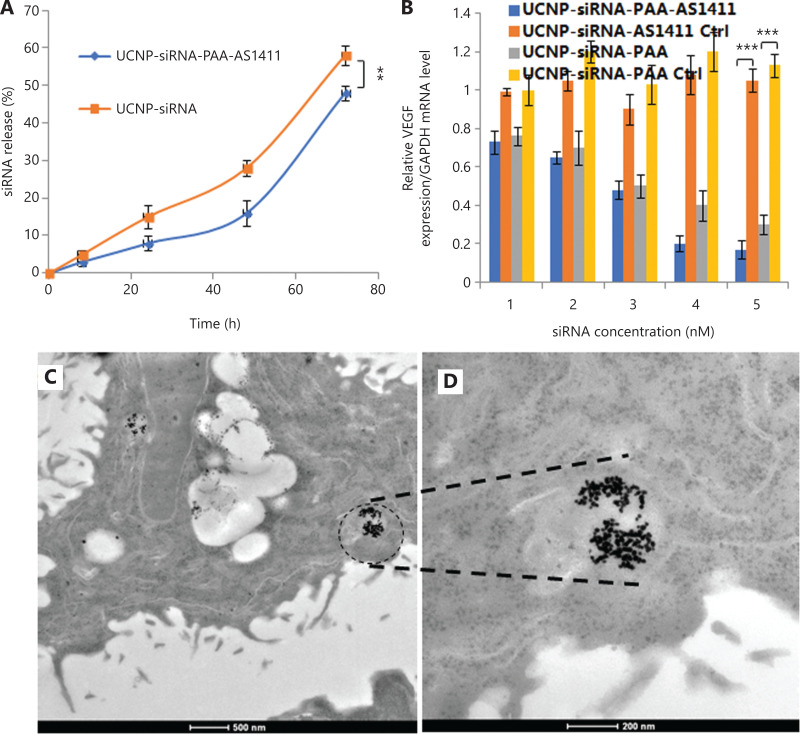
*In vitro* siRNA release, gene silence efficacy, and transmission electron microscopy (TEM) images of lung tumor tissue from treated mice. (A) The siRNA release behavior from the UCNP-siRNA-PAA-AS1411 nanocages and UCNP-siRNA; (B) The silencing efficiency of UCNP-siRNA-PAA-AS1411 and UCNP-siRNA in the NCI-H889 lung adenocarcinoma cell line. (*n* = 3; ***P < 0.005; **P < 0.01) (C, D) TEM images of NCI-H889 cells incubated with 500 µg/mL UCNP-siRNA-PAA-AS1411 nanocages.

To further develop the applications of UCNP nanocages for tumor imaging and therapy, the stability of UCNP nanocages in bioenvironmental conditions, the siRNA release behavior, the gene silence efficacy in the presence of MMP-2, the tumor cell targeting ability, and the cell apoptosis behavior were determined (**Figure 3**). To investigate UCNP nanocage stability and to evaluate the degradation times of siRNA transported by UCNP nanocages in a biological environment, cell culture medium containing 10% serum was selected for incubation of UCNP nanocages. **Supplementary Figure S1** shows that the first 24 h siRNA cumulative release of UCNP-siRNA-PAA-AS1411 was approximately 8%, when compared to that of 15% of the UCNP-siRNA. The cumulative release of siRNA of the UCNP-siRNA-PAA-AS1411 group increased to 16% over the first 48 h, with an average rate of 0.3%/h. During the 48–72 h, the average release was 1.3%, which was much lower than the UCNP-siRNA group. These results helped us to predict the release of siRNA during transit to the tumor site. The results showed that only 8% of the siRNA was released during the first 24 h, which increased our confidence that the structure of the UCNP nanocage protected the siRNA from degradation before arriving at the tumor site. In addition, the efficacies of UCNP-siRNA and UCNP-siRNA-PAA-AS1411 to silence VEGF was investigated in NCI-889 cancer cells, which showed that both groups exhibited a robust knockdown in a dose-dependent manner. As much as 85% silencing was observed at the mRNA level by qPCR at a dose of 15 nM UCNP-siRNA-PAA-AS1411 after 72 h of incubation.

We next investigated the cellular uptake in NCI-H889 human lung adenocarcinoma cells of both UCNP-siRNA-PAA-AS1411 and UCNP-siRNA. Ultrastructural TEM images showed that UCNP-siRNA-PAA-AS1411 was phagocytosed by NCI-889 cancer cells, and was mainly localized in the endosomes or lysosomes. Confocal laser scanning microscopy was used to provide visual evidence of cellular uptake and the theranostic potential of UCNP-siRNA-PAA-AS1411 and UCNP-siRNA nanoparticles. NCI-H889 human lung adenocarcinoma cells were incubated with Cy5-modified UCNP-siRNA-PAA-AS1411 and UCNP-siRNA at 37 °C for 1 h, then the nuclei were stained with Hoechst 33342 for 15 min before observation using the a Leica TCS SP8 microscope. An apparent uptake of UCNP-siRNA-PAA-AS1411 was observed after 1 h of incubation, which was more efficient than that of UCNP-siRNA, as shown by the stronger fluorescence intensity. The results confirmed the hypothesis that nanoparticles modified with AS1411 effectively improved the tumor targeting of UCNP nanoparticles.

We next investigated the antitumor efficacy using flow cytometry with annexin V-fluorescein isothiocyanate/propidium iodide double-staining to determine the degree of apoptosis induced by VEGF siRNA delivered by UCNP nanocages. NCI-889 cells were incubated with UCNP-siRNA and UCNP-siRNA-PAA-AS1411 with the MMP-2 enzyme at 37 °C for 24 h. Maximal apoptosis was observed in cells treated with UCNP-siRNA-PAA-AS1411. The early and late apoptosis subpopulations were 45.36% and 37.7%, respectively. The cell apoptosis level decreased to 55.33% (25.71% and 29.62% for early and late apoptosis, respectively) in the cell group treated with UCNP-siRNA. These results showed that the UCNP-siRNA-PAA-AS1411 improved the cellular uptake of siRNA and increased the cell apoptosis ratio in the presence of MMP-2.

### Efficient and selective *in vivo* delivery of siRNA

**Figure 4:**
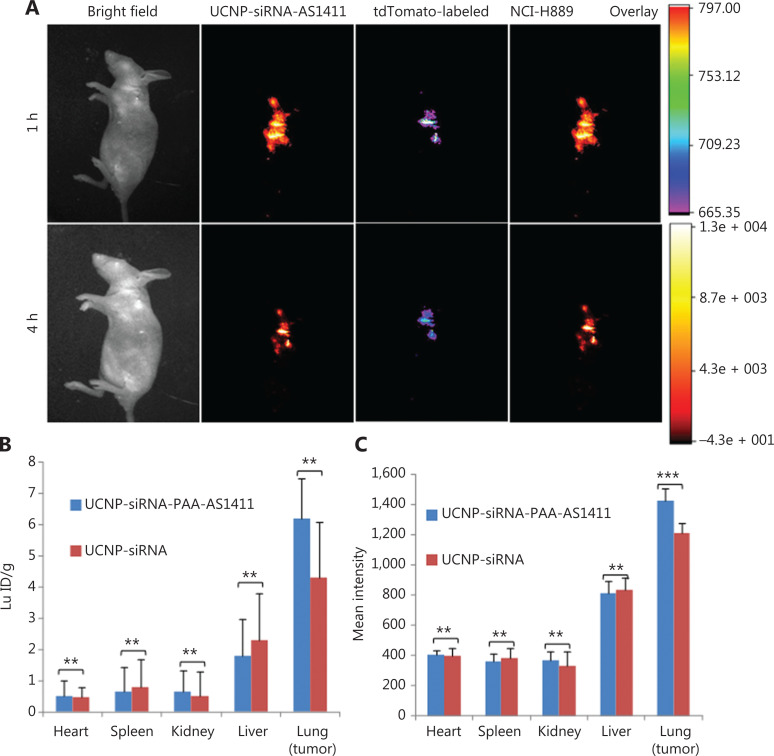
*In vivo* biodistribution studies of lung adenocarcinoma NCI-H889 tumor-bearing athymic nude mice treated with UCNP-siRNA-PAA-AS1411 nanocages. (A) Live *in vivo* bioimaging of NCI-H889 tumor-bearing athymic nude mice 1 and 4 h after inhalation with 1 mL of 1 mg/mL UCNP-siRNA-PAA-AS1411 nanocages. (B) Quantitative biodistribution of UCNP-siRNA and UCNP-siRNA-PAA-AS1411 nanoparticles in lung tumors and organs based on the region of interest analysis of the mean fluorescence intensity of each organ; (C) Quantification of UCNP distribution in organs by inductively-coupled plasma mass spectrometry. The UCNP-siRNA-PAA-AS1411 and UCNP-siRNA distributions in the liver, spleen, lung (tumor), kidney, and heart at 4 h after the inhalation-mediated localized delivery of UCNP-siRNA-PAA-AS1411/UCNP-siRNA nanocages. UCNP nanocage fluorescence: λexcitation = 980 nm, λemission = 801 ± 20 nm; tdTomato-labeled tumor fluorescence: λexcitation = 554 nm, λemission = 581 ± 20 nm; the SD (error bars) in b and c were calculated based on 6 animals (*n* = 6; ***P < 0.005; **P < 0.01) in each study group.

UCNP-siRNA and UCNP-siRNA-PAA-AS1411 were next investigated for their abilities to target delivery of anti-VEGF siRNA to lung tumors in the orthotopic murine model bearing NCI-H889 lung adenocarcinoma cells. To avoid nonspecific accumulation of UCNP nanocages by other organs and to maximize the UCNP nanocage distribution in lungs, the inhalation-mediated local delivery method was used to administer the UCNP-siRNA-PAA-AS1411 nanocages to lung tumors. For *in vivo* biodistribution imaging studies, lung adenocarcinoma NCI-H889 tumor bearing athymic nude mice inhaled 1 mL of 1 mg/mL UCNP-siRNA-PAA-AS1411, then were monitored over a time course of 24 h. **Figure 4A** shows that a strong fluorescence signal was observed, which was predominantly located at the lung site 1 h after administration. The fluorescence signal of tdTomato-labeled NCI-H889 cells was due to up-conversion fluorescence of UCNP-siRNA-PAA-AS1411 nanocages. At 4 h after administration, the fluorescence signal of UCNP-siRNA-PAA-AS1411 nanocages decreased, but overlapped with the NCI-H889 tumor signal, indicating the good tumor targeting ability of the UCNP-siRNA-PAA-AS1411 nanocages. No fluorescence signal was observed from other organs of the mice. Inductively-coupled plasma mass spectrometry (ICP-MS) showed that both UCNP-siRNA and UCNP-siRNA-PAA-AS1411 were almost exclusively concentrated in lung (tumor) tissues, with 5-fold and 10-fold increases for UCNP-siRNA and UCNP-siRNA-PAA-AS1411, respectively, compared with other organs. Only minimal accumulation was observed in other organs, such as the intestine, liver, heart, spleen, and kidney. The quantitative biodistribution of UCNP-siRNA-PAA-AS1411 nanocages was also investigated by region of interest (ROI) analyses of the mean organ fluorescence intensity. In a similar manner, the mean fluorescence intensity of the UCNP-siRNA-PAA-AS1411/UCNP-siRNA in the lung (tumor) was much stronger than that of other organs, with 15-fold and 5-fold increases, respectively. The above described results showed that UCNP-siRNA-PAA-AS1411 was primarily concentrated in lung (tumor) regions after inhalation-mediated local administration, and that minimal amounts of UCNP nanocages diffused to other organs. These results encouraged us to continue the UCNP-siRNA-PAA-AS1411 gene therapy experiments of lung cancer using the inhalation-mediated local delivery method.

### Silencing VEGF inhibited tumor growth and prolonging the survival of mice

**Figure 5:**
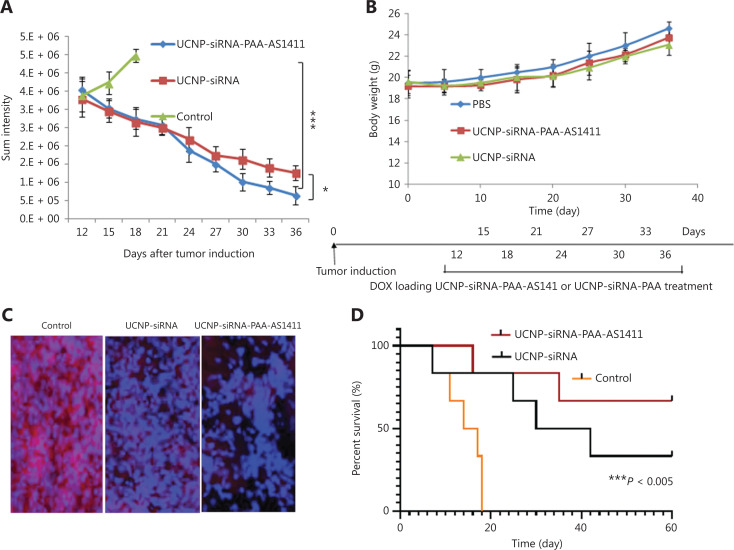
Silencing vascular endothelial growth factor (VEGF) inhibited tumor growth and prolonged the survival of mice. (A) Quantification of tumor size based on region of interest analysis of the sum fluorescence intensities of tumor tissues. (B) Weight change curve of mice treated with UCNP-siRNA-PAA-AS1411 and UCNP-siRNA during treatment. The data are expressed as the mean ± SD (*n* = 6; *P < 0.05; ***P < 0.005); (C) Lung (tumor) VEGF immunohistochemical staining showed a significant decrease in VEGF expression for the UCNP-siRNA-PAA-AS1411-treated group; (D) Kaplan-Meier survival curves of mice treated with UCNP-siRNA-PAA-AS1411, UCNP-siRNA, and the untreated control group (*n* = 6; ***P < 0.005).

Having confirmed the gene silencing effect and tumor targeting ability of the UCNP-siRNA-PAA-AS1411 nanocages, we assumed that the UCNP-siRNA-PAA-AS1411 could effectively hinder lung tumor growth through silencing the VEGF protein in NCI-H889 lung tumor cells, thus prolonging the survival of mice. Accordingly, athymic nude mice bearing lung adenocarcinoma NCI-H889 tumors were treated with both UCNP-siRNA and UCNP-siRNA-PAA-AS1411 nanoparticles at a dose of 1 mL (1 mg/mL) administered every 3 days for as long as 36 days. We monitored the tdTomato fluorescence signal of the NCI-H889 using a small animal imaging system to monitor the sizes and changes of tumor tissues. For the UCNP-siRNA-PAA-AS1411 group, the intensity of tumors decreased 1.5-fold after 7 days of treatment (18-day time point), when compared with the control group. The NCI-H889 lung tumor-bearing mice treated with UCNP-siRNA-PAA-AS1411 nanocages showed a tumor suppressive effect after 21 days of treatment, and an almost complete tumor regression after 36 days of treatment (**Figure 5A**). The results confirmed that UCNP nanocages effectively delivered siRNAs to tumor sites, which was more efficient in tumor inhibition than passive targeting using UCNP-siRNA only. The safety of UCNP nanocages was also investigated by monitoring the weight of the animals. All mice treated with UCNP-siRNA-PAA-AS1411 maintained a steady body weight, with only a 5% body weight change observed during treatment, suggesting that the UCNP nanocages were safe and nontoxic at the administered doses (**Figure 5B**). The lung (tumor) VEGF immunohistochemical staining showed 80% knockdown for the mice treated with UCNP-siRNA-PAA-AS1411, when compared with the control group, which was more efficient than that of mice treated with UCNP-siRNA (**Figure 5C**). The contribution of VEGF knockdown to mice survival was also investigated by monitoring the survival of mice treated with UCNP-siRNA and UCNP-siRNA-AS1411 nanoparticles. The survival of mice treated with UCNP-siRNA-PAA-AS1411 nanocages was significantly longer than the UCNP-siRNA and control groups after 36 days of treatment (**Figure 5D**).

### Toxicity of UCNP-siRNA-PAA-AS1411 nanocages

The safety of UCNP-siRNA-PAA-AS1411 nanocages was also evaluated. ICP-MS analyses showed that the UCNP-siRNA-PAA-AS1411 nanomaterials were mainly concentrated in the lung, followed by the liver and kidney within 24 h of administration. After 24 h, the concentration of UCNP-siRNA-PAA-AS1411 nanomaterials in the spleen and liver significantly decreased. At 7 days after administration, the concentration of Lu^3+^ in the lung significantly decreased. At 115 days after administration, almost no UCNP-siRNA-PAA-AS1411 signal was observed in all organs of the mice, indicating that most of the UCNP-siRNA-PAA-AS1411 nanomaterials were removed from the animals.

**Figure 6:**
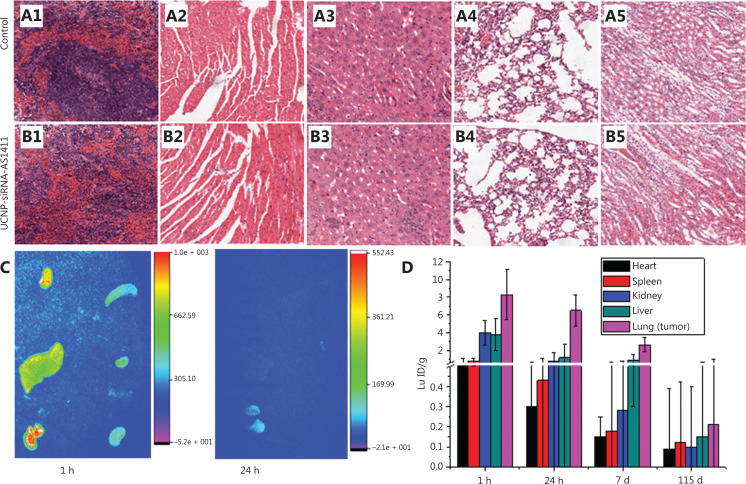
Hemoxylin and eosin-stained tissue sections from mice treated with UCNP-siRNA-AS1411, (A) for mice treated with phosphate-buffered saline, and (B) for 24 days of treatment. (A1, B1) spleen, (A2, B2) heart, (A3, B3) liver, (A4, B4) spleen, (A5, B5) kidney. Magnification: 100×. (C) The *in vitro* organ imaging of Au-siRNA-PAA-AS1411-treated mice. (D) Quantification of Lu3+ accumulation in organs by inductively-coupled plasma mass spectrometry. The data of UCNP-siRNA-AS1411 distributions in the liver, spleen, lung, kidney, and heart at 1 h and 1, 7, and 115 days after the inhalation-mediated localized delivery of UCNP nanocages. The data are expressed as the mean ± SD (*n* = 6; ***P < 0.005) in each study group.

Tissue sections from UCNP-siRNA-AS1411-treated mouse organs were also examined to evaluate potential toxicities. **Figure 6** shows that the organ morphologies of the treated mice were similar to the organs from healthy mice. Myocardial fibers showed no changes in degeneration or necrosis. The hepatic lobule structure was clear, and no obvious fat vacuoles were found in hepatic cells. The structure of the alveolar region was normal, and no interstitial fibrosis was observed. The glomerulus and tiny tubules of the kidney were normal, and hyaline changes or necrotic areas did not appear. These results confirmed that there was no significant toxicity observed in UCNP-siRNA-AS1411-treated mice, when compared with the control group.

**Figure 7:**
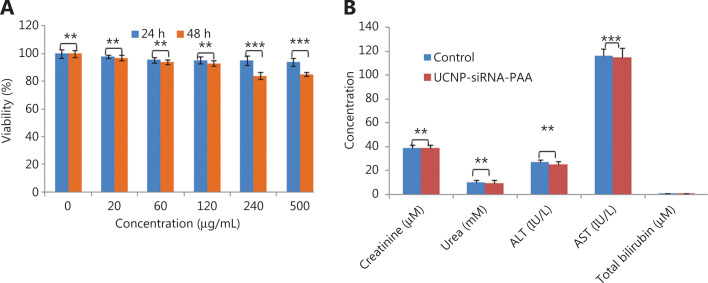
(A) The MTT assay was used to measure the cell viability after the UCNP-siRNA-PAA was given at different concentrations (0–500 µg/mL); (B) The serum biochemical indices of UCNP-siRNA-PAA-treated mice (115 days of treatment; *n* = 3, dose = 15 mg/kg) compared with mice without UCNP-siRNA-PAA administration (control). ***P < 0.005; **P < 0.01.

The MTT cell proliferation assay was used to characterize the effect of UCNP-siRNA-AS1411 on proliferation of the NCI-H889 human lung adenocarcinoma cell line. **Figure 7** shows that the cell proliferation was not significantly affected after incubation in medium containing 0–500 µg/mL UCNP-siRNA-AS1411 nanocages. After 24 h of incubation, the cell survival was more than 95%. Even when the cells were incubated with UCNP-siRNA-AS1411 at a concentration of 500 µg/mL for 48 h, the cell survival was still greater than 85%. These data showed that UCNP-siRNA-AS1411 (≤ 500 µg/mL) had low cytotoxicity. Three important liver indices, including alanine aminotransferase (ALT), aspartate aminotransferase (AST), and total bilirubin, were used to evaluate the toxicity of liver function. The results showed that the expression levels of AST, ALT, and total bilirubin were similar. Two indicators of renal function, including creatinine and urea levels, were selected to test renal function injury. The results showed that there was no significant difference between the 2 groups. Taken together, these results indicated that UCNP-siRNA-AS1411 had no obvious toxicity and may be used in biological imaging research.

## Discussion

Lung cancer originates in the tissues of the lungs or the cells lining the airways of the bronchi. The disease begins with mutations of normal cells that transform into cancer cells. These cells divide and multiply at an uncontrolled rate and eventually form tumors that impede breathing and oxygenation throughout the body. Lung cancer is currently the leading cause of cancer deaths worldwide, with 1.8 million new cases being diagnosed each year. Previous studies reported successful tumor growth inhibition by therapeutic VEGF silencing, by using nanoparticles as siRNA carriers in a mouse model bearing lung tumors^23^. However, effective protection of siRNA from degradation during transit to tumors was not achieved.

**Figure 8:**
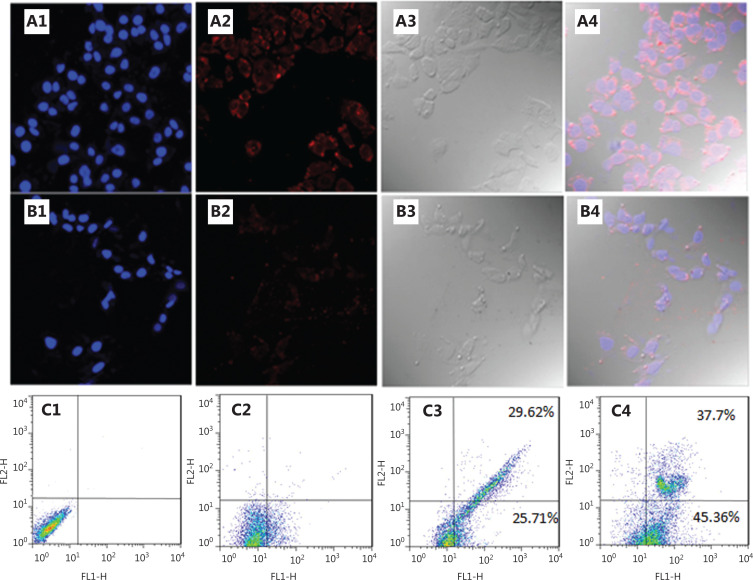
Confocal microscopy images and cell apoptosis levels of NCI-H889 lung tumor cells incubated with UCNP-siRNA and UCNP-siRNA-PAA-AS1411. (A, B) NCI-H889 cells incubated with 100 µg/mL UCNP-siRNA-PAA-AS1411 and UCNP-siRNA for 1 h. Channel 1: fluorescence signal of Cy5-modified UCNP nanocages; channel 2: Hoechst 33342 signal; channel 3: brightfield; channel 4: overlapped field. Magnification: 40×; (C) Flow cytometric analysis of NCI-H889 cells apoptosis induced by UCNP-siRNA and UCNP-siRNA-PAA-AS1411 in the presence of MMP-2 enzyme at 37 °C for 24 h.

In the present study, we designed a closed nanocage system to protect the siRNA from being degraded by nucleases. The results showed that only minimal amounts of siRNAs were released during the first 24 h in bioenvironmental conditions (**Supplementary Figure S1**), and the UCNP-siRNA-PAA-AS1411 effectively increased the cell apoptosis ratio (**Figure 8**). The results of *in vivo* distribution indicated that more siRNAs accumulated at the lung tumor site due to the AS1411 and MMP-2 modifications on the surfaces of the nanocages (**Figure 4**). The NCI-H889 lung tumor-bearing mice treated with UCNP-siRNA-PAA-AS1411 nanocages showed tumor regression after 3 weeks of treatment. We evaluated p53 expression in tumor tissues. Compared with the control group, the expression of p53 in tumor tissue treated with UCNP-siRNA-PAA-AS1411 was significantly upregulated, indicating that the inhibitory effect of VEGF on the growth of lung cancer may have been related to the p53 pathway (**Supplementary Figure S2**). The p53 is an anti-cancer gene, which can inhibit cell proliferation and negatively regulate cell growth through transactivation. To the best of our knowledge, this is the first report of the delivery of siRNAs in a relatively closed nanocage environment, which could greatly improve the delivery efficiency of siRNAs.

It should be noted that mice treated with UCNP-siRNA-PAA-AS1411 maintained a steady body weight during the treatment due to the modifications of AS1411 and MMP-2 (**Figure 5**). The modification of the MMP-2 cleavable peptide further endowed the nanocages with the ability of tumor responsive drug release, which greatly improved the drug release efficiency and reduced side effects.

## Conclusions

In summary, we have demonstrated a highly selective UCNP nanocage for effective up-conversion luminescence bioimaging and gene therapy of lung cancer. The VEGF siRNA was successfully assembled on the surface of UCNPs, and was protected by the structure of the UCNP nanocage from being degraded during transit to the tumor sites. The AS1411 on the surface of UCNP nanocages improved the targeting ability of the UCNP nanocages; the VEGF siRNA delivered by UCNP nanocages was more efficient than passive targeting when using UCNP-siRNA only. Lung tumor-bearing mice treated with UCNP-siRNA-PAA-AS1411 exhibited no body weight change, a high tumor inhibition ratio, and a longer survival time. We expect that the UCNP nanocage will contribute to the development of siRNA therapeutics for lung cancer. The structure of the UCNP nanocage could also be applicable to treat other types of cancers.
