# Supplementary material for: Dual-targeted lung cancer therapy via inhalation delivery of UCNP-siRNA-AS1411 nanocages
Source: Cancer Biol Med. 2021 Aug 24;19(7):1047–60. doi: 10.20892/j.issn.2095-3941.2020.0416 (PMC9334765; doi:10.20892/j.issn.2095-3941.2020.0416)
Supplement: Supplementary file 1 [file cbm-19-1047-s001.pdf]

Supplementary materials

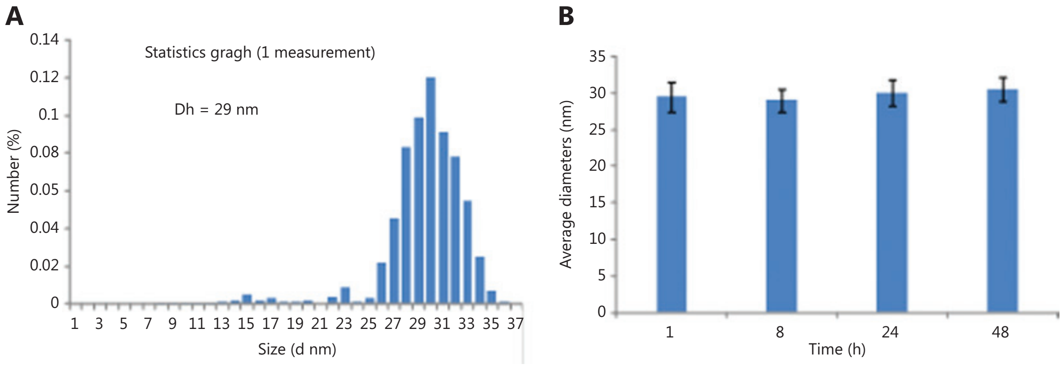

Figure S1 (A) The dynamic light scattering (DLS) curve of Au-siRNA-PAA-AS1411 in cell culture medium containing 10% serum showed the average size of nanocage was 29 nm. The size measured by DLS was larger than the size measured by transmission electron microscopy (TEM), which was due to the hydration effect and the shrinkage of nanoparticles in a drying state during TEM sample preparation; (B) The average diameter of Au-siRNA-PAA-AS1411 nanocages in cell culture medium containing 10% serum at different time points.

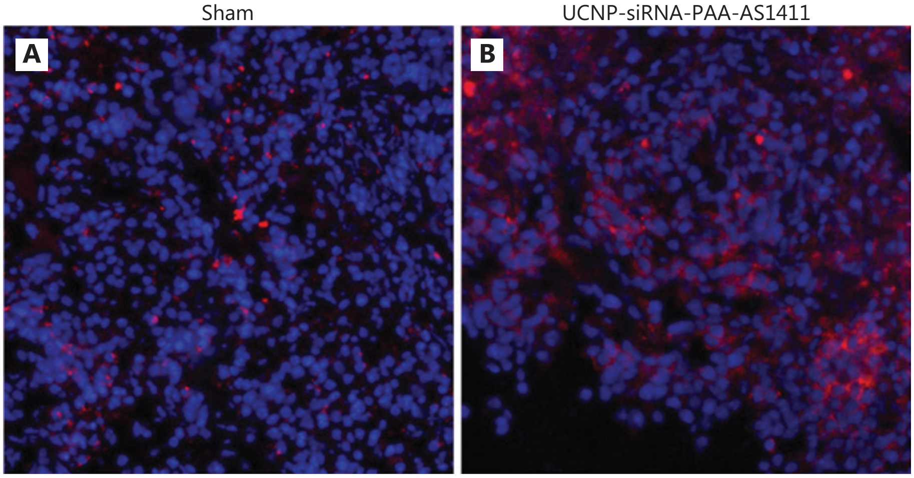

Figure S2 Lung (tumor) p53 immunohistochemical staining shows a significant increase in p53 expression for the UCNP-siRNA-PAA-AS1411-treated group.
